# Progressive cyanosis following Kawashima operation: slow resolution after redirection of hepatic veins

**DOI:** 10.1186/1749-8090-8-67

**Published:** 2013-04-05

**Authors:** Signe Holm Larsen, Kristian Emmertsen, Jesper Bjerre, Vibeke Elisabeth Hjortdal

**Affiliations:** 1 Department of Cardiology, Aarhus University Hospital, Brendstrupgaardsvej, 8200 Aarhus N, Denmark; 2 Department of Pediatrics, Aarhus University Hospital, Brendstrupgaardsvej, 8200 Aarhus N, Denmark; 3 Department of Cardiothoracic surgery, Aarhus University Hospital, Brendstrupgaardsvej, 8200 Aarhus N, Denmark

**Keywords:** Congenital heart surgery, Pulmonary arteriovenous malformations, Fontan, Left atrial isomerism

## Abstract

Progressive cyanosis often develops following Kawashima operation in patients with left atrial isomerism, interrupted inferior vena cava and hepatic veins draining to the atria. Knowledge on the timing and extend of resolution following hepatic venous redirection is sparse. A girl developed progressing cyanosis following Kawashima operation at the age of ten months. Arterial oxygen saturations at rest dropped to 60-65%. Surgical redirection of hepatic veins into the cavopulmonary circulation at the age of three years had no immediate effect. However, arterial oxygen saturations increased gradually over nine months to 90-93% and 95-100% after three years.

## Background

Patients with functional univentricular hearts, left atrial isomerism and azygos continuation of the inferior vena cava are usually surgically palliated by cavopulmonary anastomosis between one or two superior vena cavas to the pulmonary artery, the Kawashima operation. The hepatic veins remain connected to one or both left atria and thus to the systemic circulation, which in itself will cause some arterial desaturation. However, in many patients arterial desaturation is progressive causing cyanosis and functional limitation. When veno-venous collaterals from the systemic to the hepatic veins are excluded, progressive cyanosis is attributed to development of multiple microscopic pulmonary arteriovenous malformations [1,2]. It is hypothesized that one or more unknown hepatic factors from the hepatic circulation need to flow directly to the pulmonary circulation in order to suppress a genuine tendency to develop arteriovenous malformations in the lungs [3,4]. A recent study [5] suggests that inhibition of endostatin may play an important role in increased angiogenesis and formation of arteriovenous malformations. Several reports [6-9] indicate that cyanosis resolves following surgical redirection of hepatic veins into the cavopulmonary circulation. This operation is usually performed through a median sternotomy using cardiopulmonary bypass. However, an off-pump technique through a lateral thoracotomy has been described [10]. The literature on the timing and extent of resolution following redirection of the hepatic veins is sparse.

In order to demonstrate this may be a lengthy process, we report our experience of redirecting hepatic flow in a patient with left atrial isomerism and review the current literature.

## Case presentation

A newborn girl was diagnosed with dextrocardia, left atrial isomerism, azygous continuation to a solitary left sided superior vena cava, hepatic veins draining to the left sided atrium, unbalanced atrioventricular septal defect, double outlet right ventricle and valvar pulmonary stenosis. Initially the patient was surgically palliated with an aortopulmonary shunt. Kawashima operation with closure of the shunt was performed at the age of ten months. Postoperatively resting saturations were initially 85-90%, but declined over the next two years to 60-65%. Cardiac catheterization showed an unobstructed left-sided cavopulmonary anastomosis, low pulmonary artery pressures and absence of significant veno-venous collaterals. No macroscopic arteriovenous malformations were present. At the age of three years, the hepatic veins were excised from the atrium with a cuff of atrial tissue, anastomosed to a GORE-TEX® tube graft which was placed along the left side of the heart and anastomosed end-to-side to the confluent pulmonary arteries facing the superior cavopulmonary connection. The operation was done through a median sternotomy using cardiopulmonary bypass on beating heart. Postoperatively oxygen saturations were initially 60-70% but increased to approximately 85% after four weeks, 90-93% after nine months and 95-100% after three years, when she was last seen.

Pulmonary arteriovenous malformations are a common finding following the classic cavopulmonary shunt i.e. superior vena cava to right pulmonary artery [11]. However, the prevalence following total cavopulmonary connection (TCPC) has been described as relatively low [12,13], except for patients with left atrial isomerism in whom the hepatic venous flow is not incorporated directly in the TCPC. In these patients pulmonary arteriovenous malformations develops in 21-75 percent [3,7,9,14]. The large range in patients developing pulmonary arteriovenous malformations may be a combination of lack of early awareness of the problem, a wide range in the clinical presentation, additional venous-venous collaterals and a time factor, since these malformations take several years to develop. Table 1 shows a summary of the current literature with focus on the timing of surgical redirection of hepatic venous flow and time to resolution.

**Table 1:** Summary of the current literature of arterial oxygen saturations following redirection of hepatic venous flow in Kawashima patients

**Study**	**N**	**Time from Kawashima to diagnosis/ redirection of PAVMs**	**Saturation at diagnosis**	**Saturation at early follow-up (follow-up time)**	**Saturation at last follow-up (follow-up time)**
Aidala [4]*	1	3 years	65%	70% at discharge	85% (1 year)
Baskett [15]	1	≈3 years	71%	60-80%	92% (6 months)
Brown [9]	11**	5.7±3.1 years (2-11)†	75% (64-85%)‡	ND	95±2% (93-98)† (4.1±2.3 years (1-7))†
Knight [16]	1	25 months	65-70%	70%	96% (13 months)
Kutty [19]	13	2.61 years (0.61-7.35)‡	76% (70-82%)†	ND	88.7±7.6%†
Latson [20]	1	≈14 years	50%	80% (day of operation)	>93% (2 years)
McElhinney [6]	16	3.1 years (6 months-10 years)‡	76% (65-85%)‡§	76±8% (1 week-12 months)†§	90±9%† (6.3 years (2.8-10 years))‡
Pandurangi [21]	1	72 hours	40-60%§	85%||	85% ||(48 hours)
Pike [17]	1	≈2 years	68-70%	75% (4th day), 85% (3 weeks)	94% (7 weeks), 93-94% (7 months)
Shah [8]	3	8 months (7-27)‡	ND	Range 80-84% (7-10 days)	>92% (7-12 months)
Shinohara [13]	1	5 years and 9 months	75%	70% (1 month), 80 (0.5 years)	90% (2 years)

N: Number of patients, ND: Not Described,

*Partial redirection of the hepatic veins, **10 patients operated (nine evaluated for PAVMs), †Mean ± standard deviation (range),

‡Median (range), §Systemic arterial oxygen saturation, || dies 48 hours after operation

The development and diagnosis of pulmonary arteriovenous malformations following the Kawashima operation usually takes several years [4,6,9,13,15-20], but has been described after a few days [21]. Pulmonary arteriovenous malformations are usually diagnosed when oxygen saturations are between 65 and 76% [4,9,13,15-18].

Most studies report that pulmonary arteriovenous malformations resolve slowly following surgical redirection of the hepatic flow. Resting oxygen saturations as low as 70-85% for months after the operation are common [4,6,13,15,17,18]. In general, the saturations improve over time. Studies with a follow-up of several months to a few years report saturations between 93 and 97% [8,9,15,17,18,20,22]. Saturations remaining at 85-90% have been described [4,13]. Timing of redirection of hepatic flow remains controversial. A recent study [22] indicates that the majority of patients will need redirection of hepatic flow following the Kawashima operation, in particular when followed beyond 60 months. Furthermore, there was an indication that worse outcome was related to profound desaturation before redirection, indicating a benefit of an earlier redirection [22].

## Conclusions

In patients with left atrial isomerism pulmonary arteriovenous malformations should be considered when the hepatic flow has not been included in the TCPC and saturations start to decline. Following surgical inclusion of hepatic veins recovery may take several months to occur.

## Consent

Written informed consent was obtained from the family of patient for publication of this Case report. A copy of the written consent is available for review by the Editor-in-Chief of this journal.

## Competing interests

The authors declare that they have no competing interests.

## Authors’ contributions

SHL: concept/design, interpretation, drafting article, critical revision of article, approval of article. KE: concept/design, interpretation, critical revision of article, approval of article. JB: concept/design, critical revision of article, approval of article. VEH: concept/design, interpretation, critical revision of article, approval of article. All authors read and approved the final manuscript.
